# *Bactrocera dorsalis* and Its Gut Microbiota: An Emerging Insect Model

**DOI:** 10.3390/insects17070662

**Published:** 2026-06-25

**Authors:** Qi Zhou, Xiaoxue Li, Weiwei Zheng, Hongyu Zhang

**Affiliations:** National Key Laboratory for Germplasm Innovation and Utilization for Fruit and Vegetable Horticultural Crops, Hubei Hongshan Laboratory, Institute of Urban and Horticultural Entomology, College of Plant Science and Technology, Huazhong Agricultural University, Wuhan 430070, China; zq1234@wemail.hzau.edu.cn (Q.Z.); xiaoxueli@mail.hzau.edu.cn (X.L.)

**Keywords:** *Bactrocera dorsalis*, gut homeostasis, gut microbiota, insect, tephritid

## Abstract

The gut microbiota influences host health, development, nutrition, and behavior, positioning it as a frontier research area in life sciences. *Bactrocera dorsalis* is a major agricultural pest, with a short life cycle, ease of laboratory rearing, and the availability of germ-free larvae. The gut microbiota of *B. dorsalis* is complex and relatively insensitive to environmental influences. Due to these advantages, *B. dorsalis* has emerged as a promising model organism for gut microbiota research. This review synthesizes the advantages of *B. dorsalis* as a model organism, detailing its gut structure and the composition of its microbiota across developmental stages, sexes, diets, and geographical populations—highlighting the dominance of Enterobacteriaceae as a core component. Key functional roles of gut microbiota in *B. dorsalis* are elucidated, including nutrient provisioning, regulation of development and reproduction, enhancement of environmental adaptability, behavioral modulation, pesticide resistance, and immune interactions. The mechanisms underpinning gut microbiota homeostasis, involving the host Duox/ROS system, NOX enzymes, and the Imd pathway, are also discussed. Limitations are addressed, alongside future directions for leveraging genetic tools to dissect host–microbe interplay. Furthermore, the potential applications of gut microbiota research—including probiotics for Sterile Insect Technique optimization, microbial-based attractants, and paratransgenesis for pest control—are emphasized. Collectively, *B. dorsalis* offers a platform for understanding intricate host–microbe interplay and inspires novel pest management strategies.

## Simple Summary

*Bactrocera dorsalis* is a dangerous pest of fruit crops, and knowledge of its microbiota can help develop more ecologically accurate control methods. This review discusses the advantages of *B. dorsalis* as a model organism for studying the gut microbiota and describes its gut structure and microbial community composition. It details the key functional roles of the *B. dorsalis* gut microbiota, including nutrient provision, regulation of development and reproduction, enhancement of environmental adaptability, regulation of behavior, pesticide resistance, and immune regulation. The mechanisms underpinning gut microbiota homeostasis are also discussed. Furthermore, the review points out the limitations of the research and explores potential directions for future studies on the gut microbiota.

## 1. Introduction

It is well known that multicellular animals coexist with diverse microorganisms. Microbial communities are distributed across virtually every site of the host, engaging in a wide range of biological processes [1,2,3,4]. Among these, the gut microbiota have attracted extensive research attention, largely due to the distinctive physiological architecture of the gut and its direct and regular exposure to ingested food [5]. Previous studies indicated that gut microbiota influence multiple aspects of the host, including immunity, nutrition, metabolism, reproduction, and behavior [6,7,8,9]. The association between gut microbiota and host health and disease has been the focus of extensive research. However, gut microbiota research in mammals is limited due to ethical concerns, lengthy experimental cycles, high cost, limited availability of germ-free animals, and the complex composition of gut microbiota. Consequently, suitable animal models are needed to better understand host–microbe interactions, particularly in the context of host health and disease.

Model organisms are generally characterized by low maintenance costs, high reproductive rates, short generation times, small genomes, and amenability to genetic manipulation [10,11,12]. In contrast to mammals, insects are suitable experimental organisms due to their ease of laboratory rearing, shorter experimental timelines, and less complex gut microbiota. Thus, research on the insect gut microbiota has gained significant interest. As a typical insect model, *Apis mellifera* has been widely applied in gut microbiota studies due to its accessible gnotobiotic insect line, convenient genetic modification operations, and unique social biological characteristics [13]. Mosquitoes are also commonly used model insects. However, the gut community of mosquitoes is unstable and easily influenced by the environment. *Drosophila melanogaster* (Diptera, Drosophilidae) is another classic model organism commonly used to explore insect gut microbiota [14], yet it possesses inherent limitations for investigating gut microbial homeostasis and the regulatory interplay between host gene expression and microbiota. Specifically, laboratory-reared *D. melanogaster* harbors an unstable gut microbial community, with only a limited number of bacterial taxa capable of stable intestinal colonization, and exhibits substantial individual variation in gut microbial abundance [15,16]. As phylogenetically close relatives of *D. melanogaster* within the order Diptera, tephritid flies (Diptera, Tephritidae) also belong to the order Diptera, which are similar to *D. melanogaster*, sharing similarities in feeding habits and gut structure. Compared with that of *D. melanogaster*, the gut microbiota of tephritid flies exhibits greater stability, rendering it a potential model system for gut microbiota research. As a family comprising numerous agricultural pests [17], Tephritidae are widely distributed and inflict damage on various crops, thereby resulting in significant economic losses to agriculture [18]. Over the past decade, the composition and structure of the gut microbiota of various Tephritidae species have been characterized using multiple techniques, such as culture-dependent methods, 16S amplicon sequencing, shotgun metagenomics, metabolomics, transcriptomics, and gnotobiotic experiments. The composition and structure of the gut microbiota in multiple Tephritidae species have been analyzed (Table 1). The functions of major gut symbionts have been elucidated. The functions and mechanisms of action of the gut microbiota have been more comprehensively studied in *Bactrocera dorsalis* than in other Tephritidae species. Studies on the regulatory mechanisms of gut microbial community homeostasis are more systematic. Therefore, this review focuses on recent advances in research on the gut microbiota of *B. dorsalis*.

*B. dorsalis* is an excellent model organism that can be continuously reared across multiple generations under laboratory conditions, owing to its short rearing cycle [31,32]. It is a globally significant quarantine agricultural pest with a widespread distribution worldwide. Originating in Asia, *B. dorsalis* has now invaded the Americas, Africa, and Oceania, exhibiting strong adaptability and a tendency to expand northward within its suitable range [33,34]. In 2018, the first record of *B. dorsalis* complex was reported in Italy [35]. An additional advantage is that germ-free insects can be produced without specialized sterile chambers. During oviposition, *B. dorsalis* typically coats the surface of its eggs with gut microbiota, which are then acquired by the offspring via feeding [36]. Therefore, sterile larvae can be obtained by surface disinfection of eggs and subsequent feeding with irradiated, sterilized artificial diet, thereby circumventing the confounding effects of antibiotic exposure on experimental outcomes [37,38]. Sterile larvae can be used for phenotypic observations and single-organism association assays to investigate the effects of gut microbiota on the host. A comparison of *B. dorsalis* with *D. melanogaster*, *A. mellifera*, and mosquitoes is summarized in Table 2. In addition, the sequenced and annotated high-quality genome of *B. dorsalis* will further facilitate in-depth studies of host–microbe interactions [39].

## 2. The *B. dorsalis* Gut Exhibits Compartmentalized pH Regions Shaping Microbial Niches

**Figure 1:**
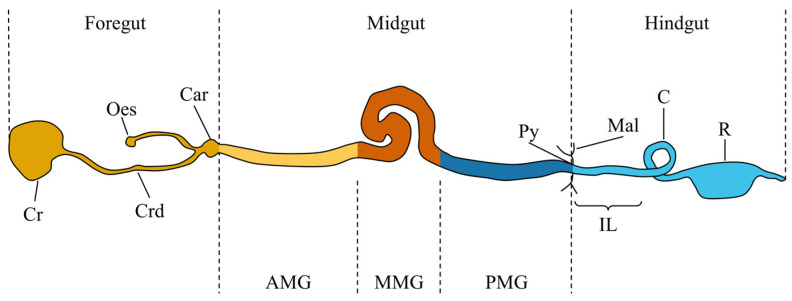
Dissected gut structure of *B. dorsalis*. The midgut of *B. dorsalis* is divided into three regions based on pH levels: AMG is neutral to slightly alkaline, MMG is acidic, and PMG is strongly alkaline. AMG, anterior midgut; MMG, middle midgut; PMG, posterior midgut; Car, cardia; Crd, cardial duct; Oes, oesophagus; Cr, crop; Py, pylorus; Mal, Malpighian tubules; IL, ileum; C, colon; R, rectum.

Compared to mammals, the intestinal structure and gut microbiota of insects are simpler [13,14,45], which is conducive to conducting research on gut microbiota. The diversity of gut microbiota is related to the structure and physicochemical properties of the insect gut. As in other insects, the gut of *B. dorsalis* is divided into three sections: the foregut, midgut, and hindgut (Figure 1). The foregut of *B. dorsalis* comprises the mouth, esophagus, and crop. As a site for food storage, the foregut harbors a high level of microbial diversity [46]. The pH of the foregut of *B. dorsalis* is acidic, which may promote the growth of acid-metabolizing bacteria. For example, *Lactobacillus* bacteria are found in high abundance in the foregut of adult *B. dorsalis* [46], which has also been observed in other *Bactrocera* species [47]. The midgut of *B. dorsalis* is primarily responsible for food digestion and nutrient absorption, and serves as the main site of colonization for symbiotic bacteria. In *Drosophila*, the midgut exhibits a trend in pH changes [48]. The midgut of *B. dorsalis* is also divided into three regions based on pH levels: the anterior midgut (AMG), the middle midgut (MMG), and the posterior midgut (PMG) [46], the pH of which is neutral to slightly alkaline, acidic, and strongly alkaline, respectively. AMG and MMG have the highest bacterial density, with Enterobacteriaceae (the primary symbiotic bacteria of *B. dorsalis*) as the most abundant in this region, whereas opportunistic pathogens are more prevalent in PMG [46]. The pH of the hindgut is alkaline, which may help regulate the function of gut microbiota. For example, the *Bacillus* in the rectum of *B. dorsalis* requires an alkaline environment to produce pheromones [49]. The differential distribution of microbiota may also be attributed to the region-specific expression of antimicrobial peptide genes in the gut, which serves to protect commensal bacteria and prevent pathogen-induced host damage [46].

## 3. *B. dorsalis* Maintains an Enterobacteriaceae-Dominated Microbiota

In recent years, advances in molecular biology and sequencing technologies have enabled researchers to analyze the gut microbiota composition of *B. dorsalis* using methods such as PCR amplification of 16S rRNA, high-throughput sequencing, and metagenomics [50,51]. *Bactrocera dorsalis* typically possesses a stable gut microbiota. Environmental factors have little effect on the composition of its primary members. This stable symbiotic relationship between *B. dorsalis* and gut microbiota provides a good framework for understanding the connection between hosts and their gut microbiota.

The gut microbial community of *B. dorsalis* exhibits high biodiversity (Table 3). At the phylum (class) level, γ-proteobacteria and Firmicutes are the dominant phyla in the vast majority of studies [40,51,52,53,54,55]. At the family level, Enterobacteriaceae are generally considered the primary bacterial group [40,51,52,53,54,55]. At the genus level, *Enterobacter* and *Klebsiella* are the most representative genera; *Citrobacter*, *Morganella*, *Providencia*, *Serratia*, *Enterococcus*, *Lactococcus* and *Achromobacter* also account for a significant proportion [40,50,53].

The composition of the gut bacterial community in *B. dorsalis* varies with host developmental stage and sex. Andongma et al. identified gut bacteria in *B. dorsalis* at different developmental stages [52]. The results revealed that gut bacterial diversity was comparable between eggs and larvae, as well as between adult males and females, whereas pupal diversity differed significantly from that of all other developmental stages. During the life cycle of *B. dorsalis*, the bacterial community transitions from dominance by Gammaproteobacteria at the larval stage to dominance by Firmicutes at the adult stage. Although gut bacterial composition varies across developmental stages, Firmicutes and Gammaproteobacteria remain the most abundant classes at every life stage, and the families Enterococcaceae and Enterobacteriaceae are also consistently present throughout the entire life cycle of *B. dorsalis* [52].

Regarding sex-based differences in gut bacterial communities, the results indicated that females harbored fewer bacterial families and exhibited lower gut microbial diversity than males. Enterobacteriaceae dominated the female community, while Orbaceae dominated the male community. Additionally, the abundance of Enterobacteriaceae in females was higher than that in males [54].

In addition to host factors, diet and geographical factors also influence the gut microbial composition of *B. dorsalis*. The gut bacterial diversity of laboratory-reared *B. dorsalis* populations is lower than that of wild populations, possibly due to the greater diversity of food sources available in the wild [51]. Kempraj et al. analyzed the ASVs of gut bacteria from adults with different fruits. Bray–Curtis dissimilarity analysis showed no significant difference in gut community composition except for one case. Compared to Bray–Curtis dissimilarities, Jaccard distances yielded far more differences between individuals. This suggests that the host has a greater influence on the secondary microbiota in the *B. dorsalis* gut [40]. This result suggests that dominant gut bacterial populations are capable of establishing stable associations with *B. dorsalis*. What is more, Enterobacteriaceae are found in all populations. This suggests that Enterobacteriaceae may play a significant role in *B. dorsalis*. The influence of geographical factors on the gut microbiota composition of *B. dorsalis* is similar to that of dietary and host factors. Members of the Enterobacteriaceae constitute the primary gut microbiota of different geographic *B. dorsalis* populations [53].

In summary, the gut microbiota of *B. dorsalis* exhibits consistency at the family level, meaning that all populations contain members of Enterobacteriaceae. Similar phenomena have also been documented in other Tephritidae species, including *Ceratitis capitata*, *Zeugodacus cucurbitae* and *Bactrocera tryoni* [24,56,57], suggesting a strong association between Enterobacteriaceae and tephritid flies. This helps elucidate the mechanisms underlying the interaction between the host and the gut microbiota. However, the absolute abundance and stability of gut microbiota communities across different populations have not been tested at the strain level. Further experimental verification is required to determine whether *B. dorsalis* from different populations exist in a core gut microbiota.

## 4. Case Studies on the Functions of Gut Microbiota in *B. dorsalis*

**Figure 2:**
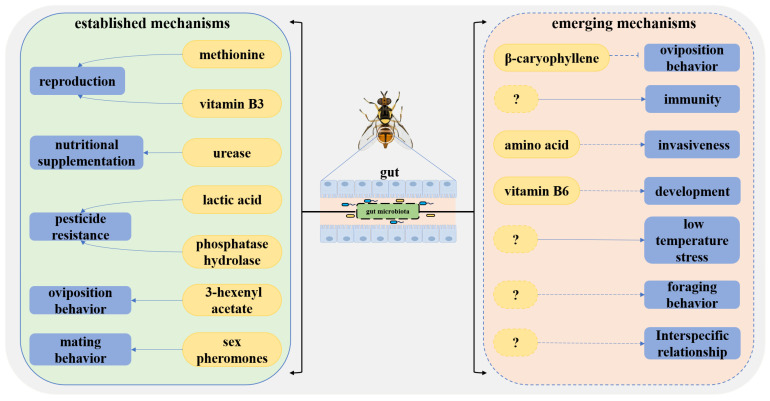
Summary of the functions of gut microbiota in *B. dorsalis*. Yellow boxes represent metabolites or enzymes produced by gut microbiota. Blue boxes represent the impact of gut microbes on the host. Solid lines represent established mechanisms. Dashed lines represent mechanisms that need to be explored. “?” represents that the metabolites produced by microorganisms are not yet clear.

The gut microbiota of *B. dorsalis* performs multiple functions within the host, including nutrient provision, development and reproduction regulation, enhancement of host adaptability, modulation of host behavior, pesticide resistance, and immune modulation (Figure 2). In this section, we summarize case studies on the functional roles of *B. dorsalis* gut microbiota to identify evidence supporting *B. dorsalis* as a model for gut microbiota research (Table 4).

### 4.1. Gut Microbiota Mediate Nitrogen Recycling and Essential Nutrient Provisioning in B. dorsalis

The gut microbiota contributes to host nutrition in *B. dorsalis* through several mechanisms, such as releasing nitrogen sources by hydrolyzing urea and synthesizing essential amino acids and vitamins. This promotes *B. dorsalis* growth, development, reproduction, and their adaptability to poor environmental conditions.

Insects frequently encounter suboptimal dietary conditions during development and reproduction, such as nitrogen deficiency [58]. Symbiotic bacteria in various phytophagous insects can directly or indirectly assist hosts in providing nitrogen to mitigate the adverse effects of nitrogen deficiency [59,60,61]. The gut microbiota of tephritid flies can provide a nitrogen source for the host through urea hydrolysis and nitrogen fixation [62,63], suggesting a positive role of gut microbiota in providing nitrogen to tephritid flies. Ren et al. reported a mechanism by which gut microbes provide nitrogen to *B. dorsalis* [64]. Nitrogenous waste accumulates continuously in citrus fruits infested by *B. dorsalis* larvae. Urease-positive gut symbiotic bacteria, including *Morganella morganii* and *Klebsiella oxytoca* in *B. dorsalis*, facilitate the host nitrogenous waste cycle by enhancing ammonia assimilation and transamination, thus supplying the host with a usable nitrogen source [64]. Herbivorous insects need to digest the polysaccharides in plants during feeding [65]. Gut microbiota play a role in the digestive process [66,67]. Saha et al. detected pectinase and xylanase activity in the gut bacteria of *B. dorsalis*, suggesting that gut microbiota may be associated with host nutrient metabolism [68].

The gut microbiota supplies the host with essential nutrients that cannot be endogenously synthesized by the host. Genes associated with amino acids and B vitamins have been identified in the genomes of various insect symbionts [69,70]. Through comparative genomic analysis of *Klebsiella* sp. BD177 isolated from *B. dorsalis* and other *Klebsiella* sp. strains, Cai et al. demonstrated that *Klebsiella* sp. BD177 is capable of specifically supplying the host with nutrients, including phenylalanine, tryptophan, methionine, folate, and riboflavin [71]. This establishes a link between gut microbiota and nutrition. However, further validation of this function requires microbial reinfection experiments.

### 4.2. Microbial Metabolites Regulate Development and Reproduction via Different Signaling in B. dorsalis

As essential associates of insects, symbiotic bacteria also modulate host development and population reproduction. In addition to providing essential nutrients to the host, the gut microbiota influences insect growth, development, and reproduction by modulating key signaling pathways. The absence of gut microbiota leads to growth and developmental delays in various insects [72,73,74,75]. Microbial loss resulting from sterility or antibiotic treatment adversely affects the development of *B. dorsalis*. Sterile larvae and pupae exhibit a significantly prolonged developmental duration, reduced larval and pupal weights, impaired immunity, and a lower adult emergence rate [76]. However, reintroduction of the gut microbiota restores these adaptive parameters. Similarly, embryonic development is also influenced by microorganisms; germ-free conditions result in a prolonged duration of this process [77]. Experiments involving single-strain associations revealed that 14 bacterial strains were capable of restoring larval development under nutrient-limited conditions. A bacterial genome-wide association study and the reinfection experiment of the mutant strain clarified the mechanism by which gut microbiota provide vitamin B6 for the host [37]. Meanwhile, a study on the reintroduction of the yeast *Hanseniaspora uvarum* into germ-free larvae showed that the gut microbiota contributes to shortening larval development duration, increasing adult wing length, and enhancing both body length and weight in larvae and adults [38]. However, the mechanisms by which gut microbiota influence *B. dorsalis* remain unknown. Future research should focus on the host response to microbiota.

Reproduction is fundamental to population sustainability, and gut microbiota significantly influence the reproductive fitness of insects [78,79]. Clearing the gut microbiota has a detrimental effect on the reproductive capacity of Tephritid [80,81]. The mechanisms by which gut microbiota affect *B. dorsalis* reproduction are established. *Enterobacter hormaechei*, a gut commensal of *B. dorsalis*, can regulate host reproduction through epigenetic mechanisms. Feeding antibiotics to eliminate gut microbiota results in arrested ovarian development in female *B. dorsalis*. *E. hormaechei*-derived methionine is required for host reproduction by modulating RNA m6A methylation of the insulin receptor gene *InR*, a key gene in the insulin signaling pathway [82]. At the same time, the nicotinic acid produced by *E. hormaechei* enhances the biosynthesis of nicotinamide adenine dinucleotide (NAD) and mitochondrial energy production, thereby activating the ubiquitin-proteasome system (UPS). The UPS further maintains the host ovarian development and reproductive capacity through the key transcription factors Lolal and decapentaplegic [83]. These studies indicate that microorganisms regulate host reproduction through a sophisticated gut–reproductive system network.

It is noted that the regulation of host development and reproduction by gut microbiota typically occurs in synergy with nutritional conditions. Under suboptimal dietary conditions, the influence of the gut microbiota on host development and reproduction becomes increasingly evident. Further investigation is warranted into the synergistic regulatory mechanisms through which nutrition and the gut microbiota mediate the development and reproduction of *B. dorsalis*.

### 4.3. Symbionts Enhance SIT Efficacy in B. dorsalis

Sterile Insect Technique (SIT) has been widely applied to the control of tephritid flies. However, while irradiation effectively induces sterility, it also inflicts concomitant physiological damage, thereby reducing the fitness and competitive performance of sterilized males [84,85]. Adding gut symbiotic bacteria to food can restore the adaptability of irradiated *B. dorsalis*. Irradiation disrupted the structure of the gut microbiota in *B. dorsalis*, resulting in a decrease in the main bacterial group Enterobacteriaceae and an increase in opportunistic pathogens [86,87]. Subsequent supplementation with a specific symbiotic strain, *Klebsiella* sp. BD177, effectively restored male mating competitiveness, flight ability, survival rate, and longevity. Furthermore, following *Klebsiella* sp. BD177 supplementation, food intake as well as hemolymph sugar and amino acid levels were increased [86]. Similarly, the addition of *Enterobacter* spp. had a positive effect on adult size, pupal weight, and survival rates under stress and nutrient-rich conditions, as well as mating competitiveness [88]. Another study showed that the addition of *Proteus* sp. to the larval diet of *B. dorsalis* significantly increased adult emergence rates, the proportion of males, and survival rates under stress conditions [89]. These studies indicate that adding probiotics to insect feed can partially mitigate irradiation-associated fitness costs. However, the study of gut microbiota mitigating the adverse effects of *B. dorsalis* only stays in the phenotypic observation; the mechanism remains to be established.

### 4.4. Symbionts Enhance Environmental Adaptation in B. dorsalis

Insects are affected by pathogens, plant defenses, and extreme temperatures during their development and reproduction. Previous studies have shown that symbionts are beneficial for insect detoxification and resistance to pathogens [90,91]. Gut microbiota in *B. minax* can help the host degrade plant secondary metabolites [92]. Emerging evidence indicates that the gut microbiota contributes to the resistance of tephritid flies to extreme temperature stress. The suitable habitat of *B. dorsalis* is continuously expanding northward. For example, the increase in average temperature is beneficial for the spread of *B. dorsalis* in Italy [93]. During invasion, colonization, and outbreak, *B. dorsalis* must adapt to various biotic and abiotic stresses. Research has demonstrated that the composition of its gut microbial community differs significantly under varying temperature regimes [94]. Notably, antibiotic treatment led to a decline in adult survival, a phenotype that was reversed by microbial supplementation. This confirms that specific bacterial strains are integral to host survival across diverse temperatures [94]. Raza et al. reported that the mechanism of *Klebsiella michiganensis* assists *B.dorsalis* resist low temperature stress. *K. michiganensis* regulates arginine and proline metabolic pathways by affecting mitochondrial function, and then regulates host resistance to cold stress [95]. Additionally, Wang et al. found that outbred *B. dorsalis* exhibited increased pupal weight, survival rate, ovarian size, and oviposition compared to inbred. There were changes in the composition of gut microbiota between inbred and outbred populations. The outbred group also displayed elevated levels of six amino acids within the gut. Supplementation with these amino acids partially rescued the compromised phenotypes in the inbred population. RNA-seq suggested that the adaptive advantages in the outbred population may be associated with the activation of the JNK-MAPK signaling pathway [96]. Further study on how symbiotic bacteria regulate the stress resistance of *B. dorsalis* is needed to elucidate the mechanisms underlying insect invasions.

### 4.5. Bacteria Modulate Behaviors Through Metabolites in B. dorsalis

The gut microbiota strongly influences the brain [97]. *B. dorsalis* exhibits complex behavior, including mating, oviposition, feeding, and attraction. Research into the effects of gut microbiota on *B. dorsalis* behavior can provide insights for behavioral studies of other insects.

Microorganisms influence the mating behavior of *B. dorsalis* by producing sexual pheromones, thereby affecting the reproduction of *B. dorsalis* populations. *Bacillus* species residing in the rectum of male *B. dorsalis* utilize glucose and threonine as substrates to synthesize the pheromones 2,3,5-trimethylpyrazine (TMP) and 2,3,5,6-tetramethylpyrazine (TTMP). The attraction effect on female flies is most significant when the concentration of TMP and TTMP is 2000 µg/mL [98]. Furthermore, female sexual attractiveness also depends on gut microbiota. Antibiotic-treated female *B. dorsalis* lose their attractiveness to males, and males mating with them exhibited reduced ejaculate volume [99]. The attraction mechanism of females to males needs further study.

Gut microbiota also plays an important role in the oviposition behavior of female *B. dorsalis*. The mechanisms by which gut microbiota regulate host oviposition behavior have been explored. On one hand, microbial metabolites can induce fruit flies to lay eggs. *B. dorsalis* females can vertically transmit *Citrobacter* sp. (CF-BD) from their gut to the egg surface. Within the host fruit, CF-BD produced the volatile compound 3-hexenyl acetate (3-HA). Female ovipositors received 3-HA and developed an oviposition preference for that fruit. The increase in 3-HA concentration led to an upward trend in EAG response of the ovipositors [100]. On the other hand, microbiota may also induce oviposition aversion. *Providencia* sp. and *Klebsiella* sp. carried on the surface of *B. dorsalis* eggs can infect host fruits. The content of β-caryophyllene in fruits infected with bacteria is higher than that in uninfected fruits (about 150 mg/mL). This avoids female flies laying eggs [101]. This microbe-mediated oviposition preference in *B. dorsalis* may help females select suitable developmental sites for their offspring.

Gut microbiota is associated with the foraging decisions of fruit flies. Under nutrient-limited conditions, germ-free larvae exhibited a preference for amino acid-rich food and shorter foraging decision times [20]. Reinfection with gut symbionts may enhance female reproductive fitness and survival rates by inducing *B. dorsalis* to make beneficial foraging decisions [102]. *B. dorsalis* foraging behavior is associated with neuropeptides [103]. Microbiota may influence host behavior through neuropeptides. The molecular mechanism by which gut microbiota influence host foraging behavior needs further study.

Microbial metabolites exert attractant effects on *B. dorsalis*. The attractant effects of symbiotic bacteria on *B. dorsalis* may assist the host in acquiring nutrients while avoiding intraspecific competition and resource depletion [104,105]. Wang et al. assessed the attractiveness of 15 strains of gut bacteria to adult *B. dorsalis* through laboratory and field bioassays. The results showed that all bacterial strains were more attractive to *B. dorsalis* than sterile culture medium, with *Bacillus cereus* and *Enterococcus faecalis* exhibiting the highest attractiveness [106]. In another study, researchers used metabolomic sequencing and bioassays to confirm that L-proline is a chemotactic substance for the gut bacterium *Enterobacter cloacae* and that it exhibits synergistic effects with the sex attractant methyl eugenol in the field [107]. These studies guided the development of pest traps.

### 4.6. Gut Microbes Confer Pesticide Resistance via Detoxification Pathways in B. dorsalis

The long-term use of chemical pesticides has led to the development of pesticide resistance in pests. Symbionts may play a role in the evolution of insecticide resistance. In *B. dorsalis*, gut microbiota may enhance host pesticide resistance through two mechanisms. Firstly, gut microbes contribute to the degradation of chemical pesticides. Antibiotic treatment reduced the resistance of *B. dorsalis* to diazinon, whereas inoculation with the gut bacterium *Citrobacter* sp. (CF-BD) restored this resistance. Comparative genomics analysis revealed that phospholytic enzymes in CF-BD may break down diazinon into less toxic metabolites, thereby enhancing the host resistance [108]. However, the function of commensal bacteria needs to be verified by bacterial mutant reinfection experiments. Secondly, gut microbiota enhanced insecticide resistance by activating detoxification pathways in *B. dorsalis*. The lactic acid produced by *Enterococcus casseliflavus* and *Lactococcus lactis* induced reactive oxygen species (ROS) via the host *BdNOX5* gene, which in turn activated the CncC pathway and thereby enhanced resistance to β-cypermethrin. Concurrently, both bacteria also activate the expression of *P450* and *GST* genes [109]. The influence of symbionts on insecticide resistance has been demonstrated across various insect species [110,111,112]. *B. dorsalis* is a major agricultural pest, and studying the impact of gut microbiota on its resistance to pesticides can aid in pest management.

### 4.7. Microbiota-Immune Crosstalk Coordinates Defense Responses in B. dorsalis

Significant progress has been made in research on the intestinal defense system, particularly regarding its role in preventing pathogen invasion and maintaining gut homeostasis [113]. However, the relationship between immunity and microbiota is not unidirectional. The gut microbiota also influences the host immune response. In *B. dorsalis*, reinfection of gut bacteria enhanced the antibacterial activity and phenoloxidase activity of germ-free larvae [76]. Research by Bai et al. has revealed a novel mechanism by which gut microbiota modulate the immune response to *B. dorsalis*. Antibiotic treatment of *B. dorsalis* led to a decrease in Duox-induced ROS levels, which in turn damaged the peritrophic matrix (PM) and subsequently activated the Imd signaling pathway. Supplementing the gut microbiota was able to reverse this effect [114].

### 4.8. Specific Symbionts Modulate Natural Enemy Interactions in B. dorsalis

Symbionts modulate interactions between host and other species, including interactions between insects and their natural enemies [115]. In other species, symbionts are believed to confer benefits to their hosts by strengthening host defense mechanisms, consequently reducing the incidence of attacks by natural enemies [116,117]. In *B. dorsalis*, different gut symbionts exerted differential effects on parasitoid fitness. *L. lactis* reduces the fecundity of parasitoids, whereas *Providencia alcalifaciens* enhances it [118]. Research on the interactions between *B. dorsalis* and its natural enemies contributes to the application of integrated pest management strategies. However, the mechanisms underlying the interaction between gut microbiota and the fruit fly-parasitoid relationship require further investigation.

**Table 4:** Effects of *B. dorsalis* gut microbiota on host and its mechanisms.

Function	Microbial Taxon/Strain	Experimental Approach	Phenotype	Mechanism	Strength of Evidence	Reference
Provide nitrogen	*Morganella morganii* and *Klebsiella oxytoca*	Metagenomics, metatranscriptomics sequencing technologies and in vitro verification tests	Promote urea hydrolysis	Nitrogenous waste recycling	In vitro validation	[64]
Polysaccharide degradation	PSG1 and PSG3	In situ and in vitro assay of enzymes	Promote pectin and xylan hydrolysis	-	Correlation analysis	[68]
Supply of amino acids and B vitamins	*Klebsiella michiganensis* BD177	Whole-genome sequencing and comparative genome analysis	-	-	Correlation analysis	[71]
Influence host development	*Enterobacteriaceae cloacae*	Gnotobiotic host, genome-wide association study, and construction of bacterial mutant strains	Increase larval length and weight	Vitamin B6 biosynthesis	Strain supplementation/knockout assay	[37]
Influence host development	*Hanseniaspora uvarum*	Gnotobiotic host	Shorten larval development duration; increased adult wing length; increased the body size and weight of both pupa and adult.	-	Strain supplementation assay	[38]
Promote host reproduction	*Enterobacter hormaechei*	RNAi; construction of bacterial mutant strains; western blot; dot blot; UHPLC−MS/MS; RNA sequencing (RNA-seq) and Methylated RNA Immunoprecipitation-m6A-sequencing (MeRIP-m6A-seq) analysis	Contributes to host ovarian development and egg laying	Methionine- RNA m6A methylation- insulin receptor	Multi-system cross validation	[82]
Promote host reproduction	*Enterobacter hormaechei*	RNAi; construction of bacterial mutant strains; western blot; LC–MS/MS analysis; Prokaryotic expression; Chromatin immunoprecipitation; proteomic and ubiquitinome mass spectrometry	Contributes to host ovarian development, egg laying and egg hatching	Nicotinic acid- ubiquitin–proteasome system- Lolal-dpp	Multi-system cross validation	[83]
Enhance SIT efficacy	*Klebsiella oxytoca* BD177	Radiation treatment and behavioral experiment	Restore the mating competition, longevity, flight parameters, food intake, levels of sugar and amino acids in the hemolymph of IR male flies.	-	Strain supplementation assay	[86]
Enhance host environmental adaptation.	*Klebsiella michiganensis* BD177	RNAi	Increase the survival rate of the host under low temperature stress	Arginine and proline metabolism pathway	Multi-system cross validation	[95]
Influence host mating behavior	*Bacillus* sp.	GC-MS, GC-EAD analysis	Enhance attraction to mature virgin females	Produce sex pheromones	Multi-system cross validation	[98]
Influence host oviposition behavior	*Citrobacter* sp. (CF-BD)	Fluorescence in situ hybridization (FISH), scanning electron microscopy (SEM), competitive binding assays in vitro, RNAi and EAG analysis	Oviposition preference	Olfactory genes expressed in ovipositor bind to 3-hexenyl acetate	Multi-system cross validation	[100]
Enhance host pesticide resistance.	*Citrobacter* sp. (CF-BD)	FISH, GC-MS, Whole-genome sequencing and comparative genome analysis	Decrease host sensitivity to trichlorphon	Produce organophosphorus hydrolase	Multi-system cross validation	[108]
Enhance host pesticide resistance.	*Enterococcus casseliflavus*, *Lactococcus lactis*	Dual-luciferase reporter gene assay and RNAi	Decrease host sensitivity to β-cypermethrin	Lactic acid- NOX5- ROS- CncC pathway	Multi-system cross validation	[109]
Affect host immunity	Gut commensal bacteria	RNAi, TEM and FITC-dextran staining	Maintaining PM structural homeostasis	Duox- ROS-PM-Imd pathway	Multi-system cross validation	[114]

## 5. Host Immune Mechanisms Maintain Microbiota Homeostasis in *B. dorsalis*

The host immune system serves as a key endogenous factor that shapes and maintains the structure of the gut microbiota. The host immune system does not simply defend passively against microbes; it actively shapes the microbial community to maintain a beneficial dynamic balance. Over the course of long-term evolution, the insect gut has developed unique defense systems to resist microbial invasion, including physical barriers, ROS mediated by the Duox [119], NADPH oxidase (NOX) [120], and antimicrobial peptides (AMPs) produced by the immune deficiency (Imd) signaling pathway [121]. The role of the immune system in maintaining gut microbiota homeostasis has been investigated in *B. dorsalis*.

Duox/ROS plays a crucial role as the first line of defense in regulating microbial homeostasis in the gut of *B. dorsalis*. RNAi knockdown of *BdDuox* leads to increased bacterial load and diversity alongside reduced symbiotic bacteria. The resulting dysbiosis activates the Duox/ROS system, which suppresses excessive proliferation of pathogens and restores gut microbiota homeostasis [122]. Moreover, neurotransmitter signaling influences the Duox/ROS-mediated regulation of gut microbiota. Serotonin suppresses *BdDuox* expression, thereby maintaining commensal bacterial load and promoting tolerance to pathogens [123]. Tyramine, released by both symbiotic bacteria and pathobionts, induces countercurrent flow between the Malpighian tubules and the gut. However, only pathobionts can induce Duox/ROS production, and the synergistic action of the countercurrent flow together with Duox/ROS promotes bacterial elimination, thereby maintaining gut microbial homeostasis in *B. dorsalis* [124].

In addition to *BdDuox*, *BdNOX5* is also a key gene involved in maintaining intestinal ROS levels. Knocking down *BdNOX5* expression significantly reduced ROS levels in the midgut and disrupted the microbial community structure. Furthermore, knocking down *BdNOX5* induced the upregulation of *BdDuox*, thereby promoting the restoration of microbial homeostasis [125]. This indicates that *BdDuox* and *BdNOX5* coordinately regulate ROS levels in the gut of *B. dorsalis* to preserve gut microbial homeostasis.

The Imd pathway is the mechanism by which fruit flies respond to Gram-negative bacterial infections, resisting pathogen invasion through the production of AMPs. PGRP-LC, as a pattern recognition receptor, is highly expressed in the foregut, facilitating the detection and clearance of pathogens. In contrast, PGRP-LB and PGRP-SB1, acting as negative regulators, exhibit expression patterns similar to the distribution of symbiotic Enterobacteriaceae, thereby suppressing excessive activation of the Imd pathway and protecting symbiotic bacteria [46,126]. Knocking down *PGRP-LB* or *PGRP-SB1* leads to upregulation of AMP expression, alterations in gut microbial structure, reduced symbiotic bacteria, and increased opportunistic pathogens, thereby inducing dysbiosis [46,127]. Furthermore, knockdown of the *Nub* gene in the Imd pathway disrupted the composition of the gut microbiota and reduced the abundance of gut microbiota [128]. Collectively, the *B. dorsalis* Duox/ROS system, NOX, and Imd pathway regulate host gut microbial homeostasis together.

## 6. Limitations and Outlook

This paper explores the potential of *B. dorsalis* as a model insect for gut microbiota research. However, studies on the gut microbiota of *B. dorsalis* still face limitations. First, although *B. dorsalis* has a stable gut microbiota that provides a good model for understanding host–microbe interactions, the gut structure of *B. dorsalis* differs from that of other model species, and their gut microbiota vary significantly and do not function identically. Therefore, research on the gut microbiota of *B. dorsalis* cannot fully substitute for research on other model species. Second, sterilizing the egg surface and feeding sterile food can only produce germ-free larvae. It is difficult to establish recipes for all the nutrients required for *B. dorsalis* development. Therefore, studies on the gut microbiota of adult *B. dorsalis* still rely on oral antibiotics to produce sterile adults, which means that the experimental results cannot rule out the influence of antibiotics. Studies have shown that antibiotics can interfere with the development and reproduction of insects [129]. In addition, antibiotics can only eliminate bacteria, but *B. dorsalis* also contains fungi, viruses, and archaea [19,38,124]. Fungi have been proven to be beneficial for the development of *B. dorsalis* [38]. At present, there are few studies on *B. dorsalis* fungi, archaea, and viruses. The composition and function of fungi, archaea, and viruses have to be discovered. Therefore, it is necessary to improve germ-free insect technology. Thirdly, as the primary habitat for microorganisms, the gut of *B. dorsalis* has not been sufficiently studied, and the cell types present in the gut have not been identified. This lack of research on the gut has limited in-depth studies of the gut microbiota of *B. dorsalis*. Therefore, future research should prioritize the study of the *B. dorsalis* gut. Fourthly, the mechanism of gut microbiota function in *B. dorsalis* is not comprehensive. Some studies only focus on phenotype observation. Future research needs to explore the mechanisms behind phenotypes. Some genetic tools have already been established and applied to *B. dorsalis*, including the CRISPR/Cas9 system for gene knockout and RNAi for knockdown [41,130,131,132]. In future work, genetic tools can be improved to conduct a more comprehensive study of host–microbe interaction mechanisms in *B. dorsalis*. Fifth, 16S rRNA sequencing based on next-generation sequencing technology has low resolution accuracy at the genus and species levels. In addition, its detection sensitivity is relatively low, which may not accurately detect low-abundance microbial species. Therefore, future research can utilize the latest sequencing technologies to identify rare taxa that were not detected in previous studies.

Importantly, the *B. dorsalis* model offers translational potential for pest management applications. For example, probiotics can be used to counteract the adverse effects of SIT and to develop microbial-based attractants [86,106]. In addition, the gut microbiota of insects can be genetically modified to express substances that kill the host or reduce its fitness, thereby controlling pest populations. This technique is known as paratransgenesis. Notably, the effectiveness of genetically modified symbiotic bacteria has been demonstrated in controlling vector-borne diseases in mosquitoes and conservation of resource insects [133,134,135,136]. However, there have been no reports of genetically modified symbiotic bacteria in tephritid flies. Future research could focus on modifying symbiotic bacteria to target genes that are lethal to the host or reduce its fitness, thereby achieving control of pest populations.

In conclusion, we discuss the advantages of *B. dorsalis* as a model for gut microbiota research. As a critical agricultural pest, *B. dorsalis* has a wide distribution and a broad host range, which reduces its rearing costs. At the same time, the gut microbiota in *B. dorsalis* exhibits a compartmentation distribution and possesses well-developed homeostatic regulatory mechanisms. Studying the *B. dorsalis* gut microbiota not only provides general insights into host–microbe interactions but also offers new approaches for pest control.

## Figures and Tables

**Table 1:** The composition of primary gut bacteria in different life stages of Tephritid fruit flies.

Tephritids	Life Stage	Dominant Bacterial Families	Research Method	Reference
*Anastrepha obliqua*	3rd instar larvae	Acetobacteraceae, Rhizobiaceae, Erwiniaceae, Enterobacteriaceae, Alcaligenaceae, Lactobacillaceae, Rhodanobacteraceae, Leuconostocaceae	PCR-DGGE fingerprinting	[19]
	Adult	Enterobacteriaceae, Rhizobiaceae, Pseudomonadaceae, Alcaligenaceae, Moraxellaceae, Xanthomonadaceae, Acetobacteraceae, Erwiniaceae, Lactobacillaceae, Halomonadaceae		
*Bactrocera dorsalis*	Egg	Enterococcaceae, Comamonadaceae, Enterobacteriaceae, Streptococcaceae, Flavobacteriaceae, Porphyromonadaceae, Pseudomonadaceae, Moraxellaceae	454 pyrosequencing	[20]
	1st instar larvae	Enterococcaceae, Comamonadaceae, Enterobacteriaceae, Streptococcaceae, Flavobacteriaceae, Porphyromonadaceae, Pseudomonadaceae, Moraxellaceae		
	3rd instar larvae	Enterococcaceae, Comamonadaceae, Enterobacteriaceae, Streptococcaceae, Flavobacteriaceae, Porphyromonadaceae, Pseudomonadaceae, Moraxellaceae		
	Pupae	Comamonadaceae, Enterobacteriaceae, Pseudomonadaceae, Moraxellaceae		
	Adult	Enterococcaceae, Enterobacteriaceae, Streptococcaceae, Flavobacteriaceae, Porphyromonadaceae		
*Bactrocera minax*	Egg, Pupae, Adult	Enterobacteriaceae, Lactobacillaceae, Enterococcaceae	454 pyrosequencing	[21,22,23]
	Larvae	Enterobacteriaceae, Lactobacillaceae, Enterococcaceae, Acetobacteraceae, Leuconostocaceae	High-throughput technologies	
*Bactrocera tryoni*	Larvae	Acetobacteraceae, Leuconostocaceae, Enterobacteriaceae, Halomonadaceae, Xanthomonadaceae	Next-generation sequencing technology	[24]
	Adult	Enterobacteriaceae, Acetobacteraceae		[25]
*Ceratitis capitata*	1st instar larvae	Enterobacteriaceae, Moraxellaceae, Streptococcaceae, Pseudomonadaceae, Methylobacteriaceae, Xanthomonadaceae	16S rDNA sequence analysis	[26,27]
	3rd instar larvae, Pupae, Adult	Enterobacteriaceae, Acetobacteraceae, Moraxellaceae, Streptococcaceae, Pseudomonadaceae, Methylobacteriaceae, Xanthomonadaceae		
*Zeugodacus tau*	Larvae	Enterobacteriaceae, Pseudomonadaceae, Enterococcaceae, Bacillaceae, Micrococcaceae, Paneibacillaceae	High-throughput technologies	[28]
	Pupae, Adult	Enterobacteriaceae, Pseudomonadaceae, Brucellaceae, Alcaligenaceae		
*Zeugodacus cucurbitae*	Larvae	Enterobacteriaceae, Mycoplasmataceae, Moraxellaceae, Enterococcaceae	High-throughput technologies	[29,30]
	Pupae	Mycoplasmataceae, Enterobacteriaceae, Caulobacteraceae, Moraxellaceae, Streptomycetaceae, Enterococcaceae		
	Adult	Enterobacteriaceae, Rhizobiaceae, Mycoplasmataceae, Streptomycetaceae, Enterococcaceae		

Data from different studies may not be comparable due to differences in methods. The proportion of primary bacteria in the host gut microbiota is at least 1%.

**Table 2:** Gut microbiota in model organisms and their research methods.

Organism	Acquisition of Microbiota	Microbiota Characteristics	Representative Species	Research Method	Reference
*B. dorsalis*	Vertical transmission via egg-surface smearing and environment	Primary microbiota less environmentally affected	Enterobacteriaceae	RNAi, gnotobiotic host and gene knockout	[37,40,41]
*D. melanogaster*	Vertical transmission via egg-surface smearing and environment	Instability and low diversity	*Lactobacillus plantarum*, *L. brevis*, *Acetobacter pomorum*	Reporter Genes in the host, gnotobiotic host, RNAi, UAS-Gal4 system and gene editing	[14]
*A. mellifera*	Social interactions	Simplification, high stability and host specificity	*Lactobacillus* Firm-4, *Bifidobacterium* spp., *Gilliamella apicola* and *Snodgrassella alvi*	Engineered strains, gnotobiotic host, RNAi and gene editing	[13,42]
mosquitoes	Environment	High variability and significant environmental impact	*Enterobacter*, *Aeromonas*	Engineered strains, gnotobiotic host, RNAi and gene editing	[43,44]

**Table 3:** The composition of gut bacteria in *B. dorsalis*.

Class	Family	Genera Detected	Life Stage
Firmicutes	Bacillaceae	*Bacillus*	Larva
Firmicutes	Streptococcaceae	*Lactococcus*	Larva, female adult, male adult
Firmicutes	Lactobacillaceae	*Lactobacillus*	Larva
Firmicutes	Exiguobacteriaceae	*Exiguobacterium*	Larva
Firmicutes	Bacillaceae	*Geobacillus*	Larva
Gammaproteobacteria	Moraxellaceae	*Acinetobacter*	Larva, male adult
Gammaproteobacteria	Pseudomonadaceae	*Pseudomonas*	Larva, female adult, male adult
Alphaproteobacteria	Caulobacteraceae	*Brevundimonas*	Larva
Firmicutes	Streptococcaceae	*Streptococcus*	Larva
Firmicutes	Leuconostocaceae	*Leuconostoc*	Larva
Firmicutes	Lactobacillaceae	*Carnobacterium*	Larva
Gammaproteobacteria	Vibrionaceae	*Enhydrobacter*	Larva
Gammaproteobacteria	Enterobacteriaceae	*Citrobacter*	Female adult
Gammaproteobacteria	Enterobacteriaceae	*Enterobacter*	Female adult, male adult
Gammaproteobacteria	Enterobacteriaceae	*Leclercia*	Female adult
Gammaproteobacteria	Enterobacteriaceae	*Serratia*	Male adult
Gammaproteobacteria	Morganellaceae	*Achromobacter*	Male adult

Reference [50].

## Data Availability

The data presented in this study are available on request from the corresponding author.
